# Estimating potassium in potato plants based on multispectral images acquired from unmanned aerial vehicles

**DOI:** 10.3389/fpls.2023.1265132

**Published:** 2023-09-21

**Authors:** YanPeng Ma, ZhiChao Chen, YiGuang Fan, MingBo Bian, GuiJun Yang, RiQiang Chen, HaiKuan Feng

**Affiliations:** ^1^ School of Surveying and Land Information Engineering, Henan Polytechnic University, Jiaozuo, China; ^2^ Information Technology Research Center, Beijing Academy of Agriculture and Forestry Sciences, Beijing, China; ^3^ National Engineering and Technology Center for Information Agriculture, Nanjing Agricultural University, Nanjing, China

**Keywords:** potato, plant potassium content, multispectral imagery, vegetation index, fraction vegetation coverage, texture feature

## Abstract

Plant potassium content (PKC) is a crucial indicator of crop potassium nutrient status and is vital in making informed fertilization decisions in the field. This study aims to enhance the accuracy of PKC estimation during key potato growth stages by using vegetation indices (VIs) and spatial structure features derived from UAV-based multispectral sensors. Specifically, the fraction of vegetation coverage (FVC), gray-level co-occurrence matrix texture, and multispectral VIs were extracted from multispectral images acquired at the potato tuber formation, tuber growth, and starch accumulation stages. Linear regression and stepwise multiple linear regression analyses were conducted to investigate how VIs, both individually and in combination with spatial structure features, affect potato PKC estimation. The findings lead to the following conclusions: (1) Estimating potato PKC using multispectral VIs is feasible but necessitates further enhancements in accuracy. (2) Augmenting VIs with either the FVC or texture features makes potato PKC estimation more accurate than when using single VIs. (3) Finally, integrating VIs with both the FVC and texture features improves the accuracy of potato PKC estimation, resulting in notable *R* ^2^ values of 0.63, 0.84, and 0.80 for the three fertility periods, respectively, with corresponding root mean square errors of 0.44%, 0.29%, and 0.25%. Overall, these results highlight the potential of integrating canopy spectral information and spatial-structure information obtained from multispectral sensors mounted on unmanned aerial vehicles for monitoring crop growth and assessing potassium nutrient status. These findings thus have significant implications for agricultural management.

## 1 Introduction

Potatoes are a significant crop with versatile uses as both a food and a vegetable. Renowned for their cold resistance, hardiness, and adaptability to various soil conditions, potatoes are extensively cultivated in China (Lu, 2015). Potatoes are a potassium-demanding crop, and the potassium content in potatoes directly influences photosynthesis efficiency and the synthesis and transport of photosynthetic products, ultimately impacting tuber development and quality (He et al., 2014; Zhang et al., 2021). Potassium deficiency in potatoes is associated with stunted plant growth, small and prematurely withering leaves, diminished photosynthetic capacity, reduced tuber size, poor quality, and low yields (Li, 2019). Conversely, excessive potassium levels can also disrupt the normal development of potatoes (Hou et al., 2013). Therefore, the efficient and accurate estimation of plant potassium content (PKC) in potatoes is of paramount importance for monitoring their growth and development and for making informed fertilizer decisions in the field. Conventional approaches for assessing PKC entail destructive sampling in the field followed by laboratory chemical analysis. However, these methods are time-consuming, labor-intensive, prone to lagging results, and reliant on the representativeness of the samples. Consequently, they primarily apply to small-scale farming plots and are challenging to extrapolate to larger areas. In contrast, remote-sensing systems exploit various sensors to capture crop canopy reflectance information from a distance, enabling non-destructive monitoring of crop growth (Battude et al., 2016; Xu et al., 2019). Valuable insights regarding crop health and nutritional status can be extracted by analyzing and processing the acquired canopy reflectance information.

Remote-sensing platforms, including ground-based hyperspectral sensors, satellite platforms, and unmanned aerial vehicles (UAVs), play a critical role in estimating crop PKC. Ground-based hyperspectral sensors are limited for providing large-scale crop-growth images and offer a limited monitoring range. Moreover, the detrimental impact on crops and the substantial consumption of human and material resources associated with this data-acquisition method hinder its application in monitoring the physical and chemical parameters of crops (Jimenez-Sierra et al., 2020). Conversely, satellite-based remote-sensing platforms face challenges in providing crop canopy images with adequate temporal and spatial resolution due to constraints, such as weather conditions (e.g., clouds, fog, and water vapor), specific revisit cycles, and large detection areas. As a result, satellite remote-sensing technology cannot satisfy the demand for real-time growth monitoring of field crops and high-accuracy estimation of crop phenotypes (He et al., 2021). In recent years, the UAV remote-sensing platform has emerged as a promising tool, boasting exceptional mobility and spatial resolution to deliver extensive, high-frequency, and precise field growth information. UAV remote-sensing technology has found widespread application in crop growth monitoring, disease surveillance, and yield estimation, offering valuable scientific and technical support for field decision management (Tao et al., 2019; Jiang et al., 2021; Lu et al., 2023).

Remote-sensing platforms based on UAVs can carry hyperspectral, multispectral, and RGB sensors (Yuan et al., 2017; Jin et al., 2018). Several studies have demonstrated the potential of UAV-based remote sensing platforms for monitoring the potassium nutrient status of crops. For instance, Lu et al. (Lu et al., 2021) developed a partial least squares model using UAV hyperspectral reflectance to estimate potassium accumulation in rice plants. Thomson et al. (Thomson et al., 2018) estimated the potassium content of forest leaves by using a partial least-squares regression model based on UAV hyperspectral reflectance. Similarly, Severtson et al. (Severtson et al., 2016) identified potassium-deficient oilseed rape using UAV-acquired multispectral and hyperspectral reflectance. Lu et al. (Lu et al., 2020) further enhanced estimation accuracy by introducing dual- and triple-band spectral indices derived from UAV hyperspectral reflectance for estimating potassium content in rice leaves. Although hyperspectral sensors provide richer spectral information, their application in agricultural production is limited by their high cost and complex data processing. Although multispectral bands are not as numerous as hyperspectral sensors, multispectral sensors contain Red and near-infraRed bands that are sensitive to vegetation, and in addition, multispectral sensors are less expensive compared to hyperspectral sensors, making them widely used in precision agriculture. Moreover, previous studies primarily relied on spectral information and vegetation indices (VIs) to estimate crop potassium content, which can be hindered by saturation in areas with dense crop canopies and the limited ability of canopy spectra to capture lower-organ information in vertically growing crops. Consequently, using canopy spectral information alone often results in inaccurate estimates of the potassium nutrient status of crops during the late stages of growth. To accurately monitor crop physicochemical parameters at single or multiple fertility stages, spectral information combined with textural details and morphological parameters such as percent cover (FVC) has been proposed to estimate crop physicochemical parameters. The FVC reflects the nutrient status of crop growth conditions to some extent and has been associated with nitrogen nutrient status in wheat, maize, rice, and potatoes (Ren, 2012; Chu, 2013; Shi et al., 2020; Fu et al., 2021; Fan et al., 2022). However, no study has yet demonstrated that morphological parameters are suitable for monitoring crop potassium nutrient status.

Texture analysis, which quantifies pixel variations within an analysis window through a grayscale distribution, has been widely used for nitrogen and biomass estimation. Combining texture information with spectral information has shown promise for significantly improving the accuracy of estimating rice canopy nitrogen content (Zheng et al., 2020). Similarly, Wang et al. (Wang et al., 2022) increased the accuracy of estimating rice aboveground biomass by incorporating canopy reflectance and UAV RGB image texture features into regression models. Additionally, Liu et al. (Liu et al., 2022a) estimated the aboveground biomass of potatoes by integrating canopy reflectance, texture information, and potato plant height derived from UAV RGB images using multiple stepwise regression and extreme machine-learning modeling. Potato growth and development differ from that of wheat, maize, and rice, with vigorous growth of stems and leaves in the early stages, followed by the transfer underground of aboveground dry matter during the late growth stages when aboveground foliage begins to deteriorate. Thus, combining VIs and the FVC, which reflect crop growth conditions, becomes crucial for estimating PKC and provides novel insights (Yue et al., 2019; Yue et al., 2021). Therefore, this study explores the performance of multispectral VIs and combinations thereof with spatial structure features for estimating potato PKC. Linear regression and stepwise multiple linear regression (SMLR) models were developed using spectral features extracted from multispectral images, FVC, and texture features. This research thus strives to develop a method for monitoring the potassium nutrient status of crops by using multispectral sensors and thereby to offer valuable insights into crop management and fertilization.

The present study has the following research objectives: (1) to assess the capacity of multispectral VIs for estimating potato PKC, (2) to examine the potential enhancement in accuracy for estimating potato PKC by integrating multispectral VIs with FVC and texture features based on the gray-level co-occurrence matrix (GLCM), and (3) to determine the optimal combination of image features to accurately estimate potato PKC. These objectives are crucial for advancing our understanding of the relationship between multispectral VIs and potato PKC estimation. Furthermore, investigating the potential synergy of combining multispectral VIs with FVC and texture features can provide valuable insights into improving the accuracy of PKC estimates. Ultimately, identifying the most effective combination of image features will contribute to developing robust and precise methods for monitoring and managing the potassium nutrient status of potato crops.

## 2 Materials and methods

### 2.1 Experimental design

**Figure 1:**
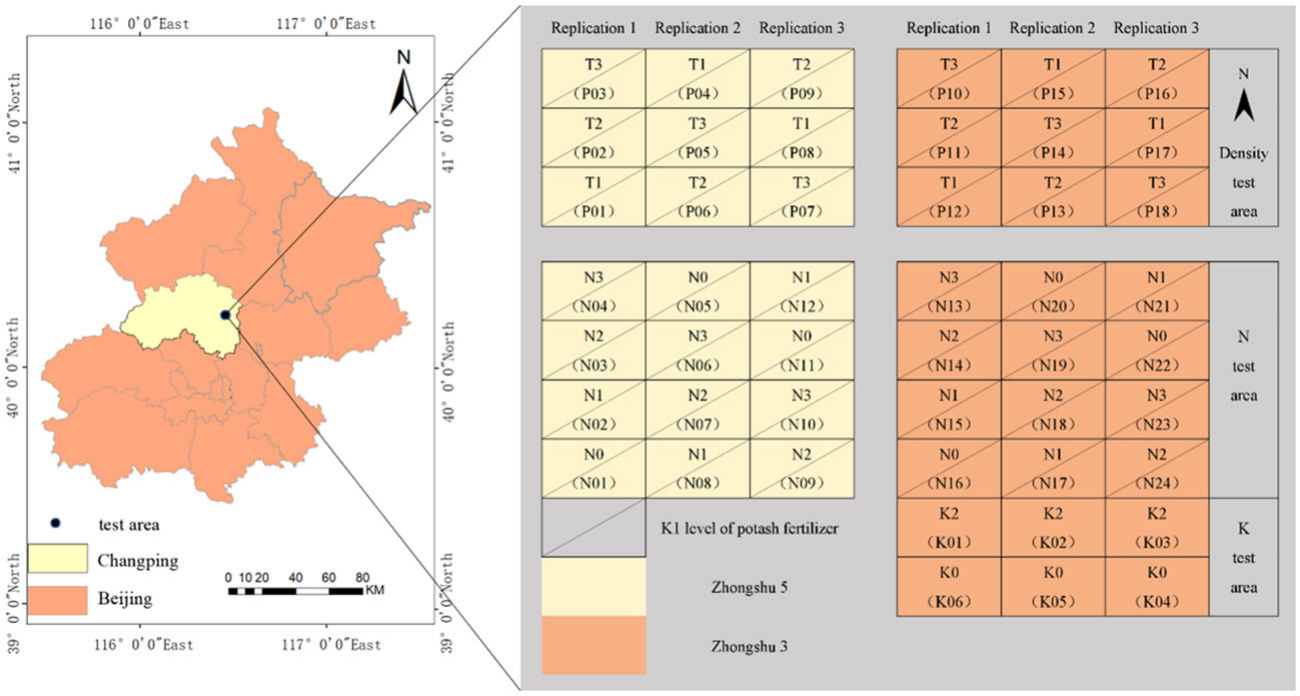
Schematic diagram of the location of the field.

The field experiment was conducted in 2019 at the National Precision Agriculture Research and Demonstration Base situated in Xiaotangshan Town, Changping District, Beijing, China. The study site is characterized by an average altitude of 36 m and a warm temperate continental semi-humid and semi-arid monsoon climate, exhibiting simultaneous rainfall and high temperatures during the same season. Potato seed tubers were sown on 28 March 2019 and harvested on 9 July 2019. Two early-maturing potato varieties, Zhongshu 5 (Z1) and Zhongshu 3 (Z2), were selected as the subjects of this study. The experiment comprised three distinct experimental zones: dense (P zone), nitrogen fertilizer (N zone), and potassium fertilizer (K zone), with each zone having three replications, as depicted in **Figure 1**. The experiment involved a total of 48 plots, each measuring 5 m × 6.5 m. Within the P zone, three different planting density levels were implemented: T1 (60 000 plants/hm^2^), T2 (72 000 plants/hm^2^), and T3 (84 000 plants/hm^2^). The N zone used four nitrogen fertilizer levels: N0 (0 kg/hm^2^), N1 (244.65 kg/hm^2^), N2 (489.15 kg/hm^2^), and N3 (733.5 kg/hm^2^). Two types of potash fertilizers were used in Area K: K0 (0 kg/hm2) and K2 (1941 kg/hm2). Notably, the planting density and nitrogen-test areas were consistently treated with the K1 level of potash fertilizer, both N and K plots were treated under T1 density. This comprehensive and meticulously designed experimental setup allows the study to investigate how planting density, nitrogen fertilizer level, and potash fertilizer level affect the PKC of the potato plants. Incorporating multiple treatments and replications ensures that robust and reliable data are generated, facilitating subsequent modeling and analysis.

### 2.2 Acquisition and preprocessing of UAV multispectral images

UAV flight operations were conducted on April 20, May 28, June 10, and June 20, 2019, to obtain digital images. To mitigate the influence of uneven illumination on crop canopy reflectance, this experiment meticulously selected clear and cloudless weather conditions during the crucial stages of potato tuber formation stage (S1), tuber growth stage (S2), and starch accumulation stage (S3). Multispectral images were acquired between 12:00 and 14:00, when the ambient light intensity is stable. Prior to the flight, the spectral reflectance calibration plate associated with the multispectral sensor was used to calibrate the brightness of individual elements within the multispectral images, ensuring accurate radiometric calibration. This study used an eight-rotor UAV equipped with a Parrot Sequoia 4-channel multispectral camera. The Parrot Sequoia camera comprises a light sensor and a multispectral sensor, enabling the acquisition of one high-resolution 16-megapixel RGB image along with four 1.2-megapixel single-band images. The UAV was operated at a flight altitude of 20 m, and both the heading overlap and side overlap were set at 80% to ensure comprehensive coverage and minimize information gaps. **Table 1** presents the built-in waveband parameters of the multispectral camera used in this study, facilitating the capture of specific spectral information relevant to the analysis of potato growth and potassium nutrient status. The rigorous control of environmental conditions and the use of a well-calibrated multispectral camera mounted on the UAV allow high-quality data to be collected, thereby ensuring the accuracy and reliability of subsequent image analysis and extracted information.

**Table 1:** Parametrization of multispectral sensor.

Band	Central wavelength (nm)	Bandwidth (nm)
Green	550	40
Red	660	40
Reg	735	10
Nir	790	40

After the acquisition of UAV data and multispectral images during each reproductive period, a series of preprocessing steps were undertaken. Initially, the multispectral images obtained from the UAV were carefully screened to eliminate images with abnormal attitudes or imaging issues. The remaining high-quality multispectral images were then imported into the DJI SmartMap software, where single-band and band-combination images were generated and saved in the TIF format. Next, by using the ArcGIS software, the experimental plots were delineated and numbered based on the predefined divisions. This spatial referencing enables accurate association of the multispectral data with specific plot locations. Subsequently, the average spectral reflectance for each plot was computed for each band using ENVI5.3 software. This process involved analyzing the multispectral data for each plot and calculating the corresponding spectral reflectance across the relevant bands. Additionally, VIs were derived from the spectral reflectance data, contributing to further analysis and interpretation of the crop’s physiological state. These steps eliminated data inconsistencies and facilitated the extraction of meaningful information from the multispectral images, enabling subsequent analysis and interpretation of the potato crop’s growth and potassium nutrient status.

### 2.3 Acquisition of ground data

Once the UAV data were collected, meticulous ground-data collection complemented the remote sensing observations. The primary focus of the ground data was to determine the potato PKC through a combined approach involving field sampling and laboratory chemical analysis. Within each experimental plot, three representative plants were carefully chosen during key developmental stages: potato tuber formation, tuber growth, and starch accumulation, following which the selected plants were collected and transported to the laboratory for further analysis. The collected plants underwent meticulous processing in the laboratory, including separating stems and leaves and thoroughly rinsing them with water. The plants were subsequently heated in a 105°C oven for 30 minutes. The temperature was then adjusted to 80°C, and the plants were dried for a minimum of 48 hours until a constant mass was achieved. Once the mass reached a steady state, the plants were weighed to determine the dry weight of each organ. Advanced laboratory techniques were employed to measure each organ’s potassium content, notably an inductively coupled plasma emission spectrometer (iCAP6300). This state-of-the-art instrument allowed for precise quantification of potassium levels in the plant samples. Finally, the PKC was calculated using


(1) $$PKC=\frac{C_{LK}\times M_{LD}+C_{SK}\times M_{SD}}{M_{LD}+M_{SD}}\times 100\%,$$

where C_LK_ is the leaf potassium content (%), C_SK_ is the aboveground stem potassium content (%), M_LD_ is the leaf dry weight (g), and M_SD_ is the aboveground stem dry weight (g).

Combining meticulous field sampling and precise laboratory analyses, this comprehensive ground data collection process allows for accurate estimation and understanding of the potato crop’s potassium nutrient status. It provides essential validation and calibration data for the UAV-based remote-sensing observations, thereby enhancing the reliability and accuracy of the study.

### 2.4 Extraction of image features

#### 2.4.1 Selection of spectral index

To construct a robust estimation model for potato PKC, nine multispectral VIs were carefully selected for each fertility period. These VIs were chosen based on an extensive analysis of previous research findings and their proven efficacy in monitoring crop potassium nutrient status, they were correlated with potato PKC at a 0.01 significant correlation level. The selected VIs, along with their respective definitions and formulas, are presented in **Table 2**.

**Table 2:** Vegetation indices used in this study.

VIs	Formula	Reference
Green	Green band reflectance	
Red	Red band reflectance	
Reg	Reg band reflectance	
NIR	Nir band reflectance	
NDVI	NDVI = (Nir − Red)/(Nir + Red)	(Carlson and Ripley, 1997)
SAVI	SAVI = 1.5×(Nir − Red))/(Nir + Red)+ 0.5)	(Huete et al., 1992)
GNDVI	GNDVI = (Nir − Green)/(Nir + Green)	(Buschmann and Nagel, 1993)
RDVI	RDVI = (Nir − Red)/(Nir + Red)^1/2^	(Broge and Leblanc, 2001)
OSAVI	OSAVI = 1.16×(Nir − Red)/(Nir + Red + 0.16)	(Rondeaux et al., 1996)
MSR	MSR = (Nir/Red − 1)/(Nir/Red + 1)^1/2^	(Sims and Gamon, 2002)
DVI	DVI = Nir − Red	(Richardson and Wiegand, 1977)
RVI	RVI = Nir/Red	(Schlerf et al., 2005)
NLI	NLI = (Nir^2^ − Red)/(Nir^2^ + Red)	(Pu et al., 2008)

#### 2.4.2 Extraction of spatial structure features

In this study, two spatial structure features, namely, FVC and the GLCM-based texture features, were used to characterize the spatial patterns of the potato crop. These features were derived from the UAV-acquired multispectral images and were crucial in assessing the potato’s growth stages (Li et al., 2004). To extract the FVC, the multispectral images corresponding to each fertility stage were processed using image element dichotomy. Initially, the normalized difference Vegetation Index (NDVI) was computed for each fertility stage by employing ENVI 5.3 software. The NDVI values were then quantified, and subsequently, the FVC was calculated according to Equation 2, as shown below:


(2) $$FVC=\frac{NDVI-NDVI_{Soil}}{NDVI_{Veg}-NDVI_{Soil}}$$

Where NDVI_Soil_ and NDVI_Veg_ represent NDVI values with a cumulative percentage of 5% and 95%, respectively.

Texture Feature Extraction: In this study comprehensive set of eight texture features derived from the GLCM was extracted from the multispectral images. These features, namely contrast (CON), second-order moments (SEC), variance (VAR), mean (MEA), correlation (COR), dissimilarity (DIS), homogeneity (HOM), and entropy (ENT), provide valuable insights into the spatial arrangement and variation of pixel intensities within the potato crop (Roujean and Breon, 1995). To obtain the texture features, GLCM calculations were performed in four directions (0°, 45°, 90°, and 135°) using a 3×3 window size for each spectral band. Each texture feature was computed individually from the GLCM matrices, capturing different aspects of the spatial patterns in the multispectral images. Furthermore, two spectral indices, namely the NDVI and the differential vegetation index (DVI), were used in this study. The NDVI is less affected by canopy spectral properties and directional effects, whereas the background signal is less affected by the DVI. Combining the advantages of both indices, the renormalized differential vegetation index (RDVI) has been widely used for estimating various physicochemical parameters of crops (Roujean and Breon, 1995; He et al., 2019). To enhance the performance of texture features for estimating potato PKC, the texture features were combined based on the RDVI. This integration allows us to leverage the benefits of RDVI and optimize the texture features’ predictive capability for accurately estimating the PKC. The RDTI is given as:Red


(3) $$RDTI=\frac{T_{1}-T_{2}}{\sqrt{T_{1}+T_{2}}}$$

where T1 and T2 stand for the extracted randomized texture features for each fertility period from the multispectral images in the r, g, b, Red, and Nir bands.

### 2.5 Model building and validation

A total of 48 datasets were acquired at different stages of potato development, including the potato tuber formation, tuber growth, and starch accumulation stages. From these datasets, 32 were selected from replicates 1 and 3 for model development, and the remaining 16 datasets from replicate 2 were reserved for independent validation, ensuring the reliability and robustness of the experimental findings. A linear regression analysis determined the relationship between multispectral VIs and potato PKC. The SMLR model was also applied to investigate the potential enhancement in PKC estimation accuracy by integrating multispectral VIs with the RDTI and FVC. To evaluate the performance of the models, the coefficient of determination (*R* ^2^) and the root mean square error (RMSE) were used as indicators of model stability and accuracy. *R* ^2^ represents the fraction of the variance in potato PKC that the model can explain, and the RMSE measures the average deviation between the predicted and actual PKC. These evaluation metrics allow for a comprehensive assessment of the model’s performance, ensuring the validity and precision of the estimated potato PKC.

## 3 Results

### 3.1 Potassium nutrient variations and correlation analysis of potatoes under different experimental treatments

**Figure 2:**
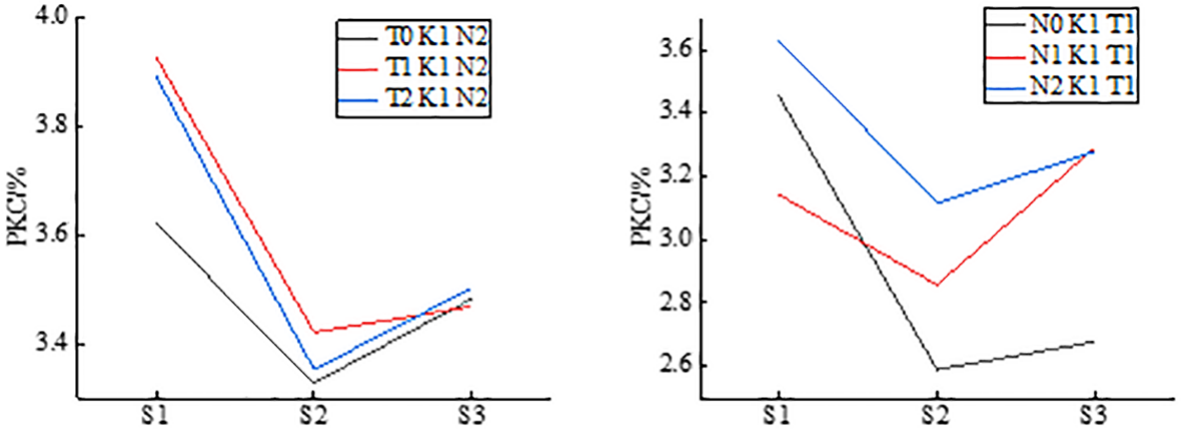
PKC as a function of growth period for potatoes under different experimental treatments.

Note: S1, S2, and S3 are the tuber formation stage, tuber growth stage, and starch accumulation periods stage, respectively; the same below.

As shown in **Figure 2**, for all experimental treatments, potato PKC decreases first and then increases with the reproductive stage, while the highest PKC content was found in all other treatments during tuber formation and continued to decline until starch accumulation, where the lowest PKC was found during tuber growth.

**Table 3** shows the results of the GLCM-based correlation analysis of texture features extracted in different directions with potato PKC. The absolute value of the correlation coefficient during the tuber formation period was 0.81 for Green_Cor in the 90° direction. The absolute values of the correlation coefficients at 0°, 45°, and 135° do not differ significantly from those at 90° at the same significance level of 0.80, 0.79, and 0.74, respectively. The largest absolute value of the correlation coefficient for the tuber growth stage was Nir_Var, with equal magnitude in all four directions, at 0.82. The largest absolute value of the correlation coefficient for the starch accumulation stage is Green_MEAN, with an equal magnitude of 0.80 in all four directions. The best-performing textural characteristics in the three reproductive stages do not differ significantly from those of potato PKC. Thus, the different orientations of the three fertility periods negligibly affect the Mean and Var texture traits in the Green, Red, Reg, and Nir bands.

**Table 3:** Correlation analysis between GLCM texture and potato PKC in different directions.

	S1_GLCM				S2_GLCM				S3_GLCM			
Plot	0°	45°	90°	135°	0°	45°	90°	135°	0°	45°	90°	135°
Green_Mean	−0.75	−0.75	−0.75	−0.76	−0.78	−0.78	−0.78	−0.78	−0.80	−0.80	−0.80	−0.80
Green _Var	−0.63	−0.63	−0.63	−0.64	−0.68	−0.68	−0.68	−0.68	−0.34	−0.34	−0.34	−0.34
Green _Hom	0.68	0.63	−0.33	0.71	0.46	0.32	0.04	0.31	0.21	0.11	0.11	0.20
Green _Con	−0.62	−0.59	−0.38	−0.65	−0.61	−0.64	−0.54	−0.66	−0.30	−0.29	−0.33	−0.38
Green _Dis	−0.64	−0.60	−0.13	−0.67	−0.56	−0.52	−0.27	−0.53	−0.22	−0.17	−0.18	−0.27
Green _Ent	−0.07	−0.21	−0.16	−0.26	−0.10	−0.13	0.02	−0.12	−0.16	−0.16	−0.16	−0.18
Green _Sec	0.11	0.22	0.16	0.29	0.06	0.09	−0.07	0.08	0.17	0.16	0.15	0.18
Green _Cor	−0.80	−0.79	−0.81	−0.74	−0.48	−0.49	−0.52	−0.38	−0.30	−0.45	0.15	0.12
Red_MEAN	−0.67	−0.67	−0.67	−0.67	−0.74	−0.74	−0.74	−0.74	−0.56	−0.56	−0.56	−0.56
Red _Var	−0.57	−0.57	−0.57	−0.58	−0.59	−0.59	−0.59	−0.60	−0.49	−0.49	−0.49	−0.49
Red _Hom	0.62	0.59	0.50	0.63	0.76	0.75	0.69	0.74	0.57	0.53	0.51	0.58
Red _Con	−0.57	−0.57	−0.54	−0.59	−0.63	−0.60	−0.55	−0.60	−0.53	−0.44	−0.46	−0.56
Red _Dis	−0.60	−0.59	−0.11	−0.62	−0.69	−0.67	−0.61	−0.67	−0.55	−0.48	−0.48	−0.57
Red _Ent	−0.49	−0.51	−0.51	−0.54	−0.78	−0.79	−0.78	−0.79	−0.56	−0.54	−0.55	−0.57
Red _Sec	0.44	0.46	0.42	0.49	0.79	0.80	0.79	0.80	0.55	0.54	0.54	0.56
Red _Cor	−0.76	−0.78	−0.77	−0.66	−0.73	−0.54	−0.71	−0.70	−0.07	−0.23	−0.34	0.13
Reg_ Mean	0.70	0.70	0.70	0.70	0.62	0.62	0.62	0.62	0.48	0.48	0.48	0.48
Reg _Var	0.54	0.54	0.54	0.54	0.68	0.68	0.68	0.67	0.48	0.48	0.48	0.48
Reg _Hom	−0.32	−0.41	−0.67	−0.40	−0.59	−0.60	−0.62	−0.61	−0.44	−0.44	−0.48	−0.47
Reg _Con	0.52	0.54	0.60	0.54	0.66	0.69	0.69	0.66	0.47	0.48	0.50	0.49
Reg _Dis	0.49	0.51	0.63	0.51	0.64	0.66	0.66	0.64	0.47	0.47	0.50	0.49
Reg _Ent	0.64	0.63	0.63	0.63	0.57	0.57	0.57	0.56	0.44	0.44	0.42	0.43
Reg _Sec	−0.63	−0.63	−0.63	−0.63	−0.56	−0.56	−0.56	−0.55	−0.43	−0.43	−0.41	−0.42
Reg _Cor	−0.74	−0.66	−0.69	−0.73	−0.25	−0.32	−0.28	−0.11	0.03	0.02	0.01	−0.18
Nir_ Mean	0.70	0.70	0.70	0.70	0.77	0.77	0.77	0.77	0.60	0.60	0.60	0.59
Nir _Var	0.63	0.63	0.63	0.62	0.82	0.82	0.82	0.82	0.63	0.63	0.63	0.63
Nir _Hom	0.56	0.28	−0.79	0.43	−0.68	−0.68	−0.68	−0.68	−0.49	−0.52	−0.55	−0.52
Nir _Con	0.48	0.71	0.83	0.55	0.81	0.81	0.80	0.81	0.61	0.63	0.65	0.63
Nir _Dis	0.30	0.64	0.83	0.39	0.78	0.78	0.76	0.77	0.57	0.61	0.62	0.60
Nir _Ent	0.62	0.57	0.59	0.54	0.61	0.60	0.61	0.60	0.44	0.43	0.40	0.42
Nir _Sec	−0.55	−0.50	−0.52	−0.49	−0.60	−0.59	−0.59	−0.58	−0.41	−0.39	−0.36	−0.38
Nir _Cor	−0.59	−0.72	−0.80	−0.57	−0.12	−0.04	−0.23	−0.02	0.20	0.06	0.01	0.06

Green_Mean is the mean texture feature of Green, and others analogously.

**Figure 3:**
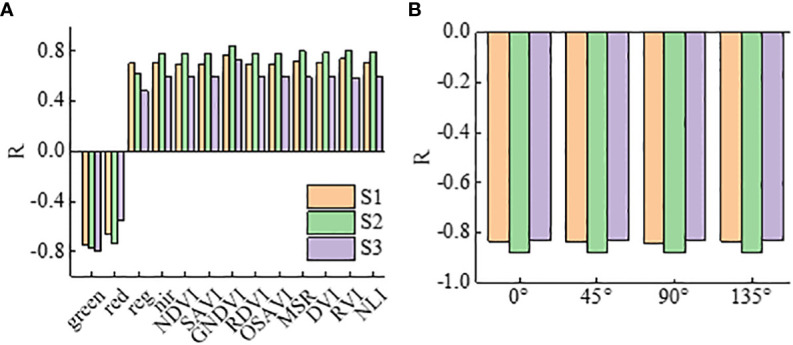
Correlation analysis: **(A)** is the correlation between multispectral VIs and potato PKC, and **(B)** is the correlation between RDTI and potato PKC composed of texture features based on different orientations of GLCM.

The multispectral VIs and the RDTI composed of texture features based on GLCM with different orientations were correlated with potato PKC at each fertility stage of potato growth, and the results are shown in **Figure 3**. The correlation between the VIs constructed from the original band reflectance and PKC reach a significant 0.01 correlation level in all three growth stages, with the absolute values of correlation ranging from 0.46 to 0.78, respectively, with the GNDVI being the highest. As shown in **Figure 3B**, the correlation between the RDTI and potato PKC based on texture features of different orientations of GLCM does not vary significantly, and the correlation with multispectral VIS is comparable, indicating that the RDTI based on texture features of different orientations of GLCM is feasible for estimating potato PKC.

### 3.2 Multispectral VIs for estimating potato PKC

**Figure 4:**
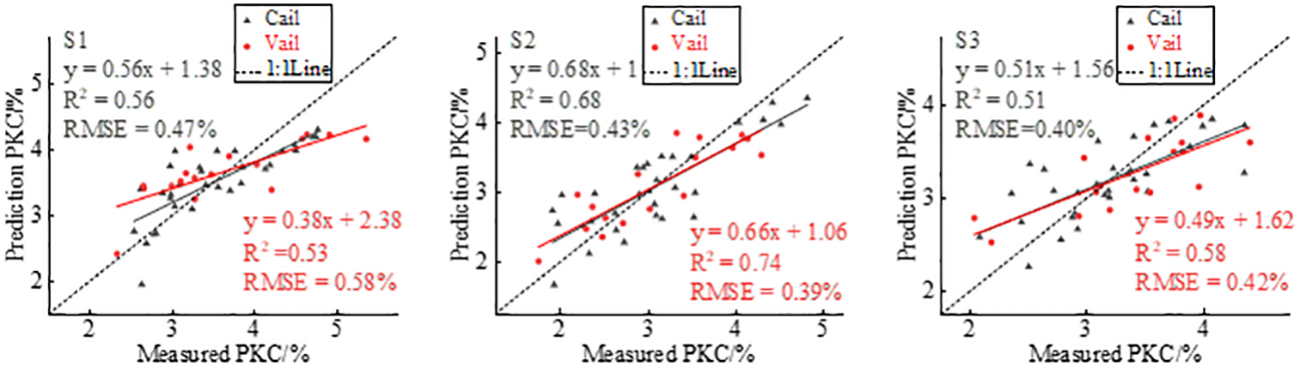
Predicted PKC plotted versus measured PKC to show effect of GNDVI modeling and verification.

In this study, based on multispectral images to extract the canopy reflectance of potatoes at three reproductive stages, nine VIs were calculated and linear regression modeling was used to estimate potato PKC. **Table 4** shows the estimated results. The *R* ^2^ range is 0.40–0.56 and the RMSE range is 0.47%–0.56% for tuber formation, 0.56–0.68 and 0.43%–0.50% for tuber growth, and 0.42–0.51 and 0.40%–0.42% for starch accumulation, respectively. The scatter plots of actual and predicted potato PKC values modeled by GNDVI have *R* ^2 = ^0.56, 0.68, and 0.51 and the RMSEs are 0.47%, 0.43%, and 0.40%, respectively. *R* ^2^ is greater than 0.50, indicating that GNDVI could reflect the potato PKC status to some extent. GNDVI estimation potato PKC modeling and validation results are shown in **Figure 4**. Each VI model estimates potato PKC with the highest *R* ^2^ at the tuber growth stage.

**Table 4:** Multispectral VIS modeling estimation of potato PKC in three growth stages.

VIS	S1		S2		S3	
	*R* ^2^	RMSE (%)	*R* ^2^	RMSE (%)	*R* ^2^	RMSE (%)
DVI	0.42	0.55	0.57	0.50	0.42	0.42
MSR	0.43	0.54	0.62	0.47	0.44	0.41
NDVI	0.40	0.56	0.56	0.51	0.43	0.42
NLI	0.42	0.55	0.59	0.49	0.43	0.42
OSAVI	0.41	0.55	0.56	0.50	0.43	0.42
RDVI	0.41	0.55	0.57	0.50	0.43	0.42
RVI	0.44	0.54	0.65	0.45	0.44	0.41
SAVI	0.41	0.55	0.57	0.50	0.43	0.42
GNDVI	0.56	0.47	0.68	0.43	0.51	0.40

### 3.3 Multispectral VIs combined with spatial structure features to estimate potato PKC

To test whether the multispectral VIs fusing the spatial structure features of multispectral images can improve the accuracy of estimating potato PKC, this study extracts the FVC of potato S1–S3 based on multispectral image NDVI using image element dichotomy and also calculates the RDTI based on GLCM extracting texture features in the 0°, 45°, 90°, and 135° directions.

#### 3.3.1 Multispectral VIs combined with FVC for estimation of potato PKC

**Figure 5:**
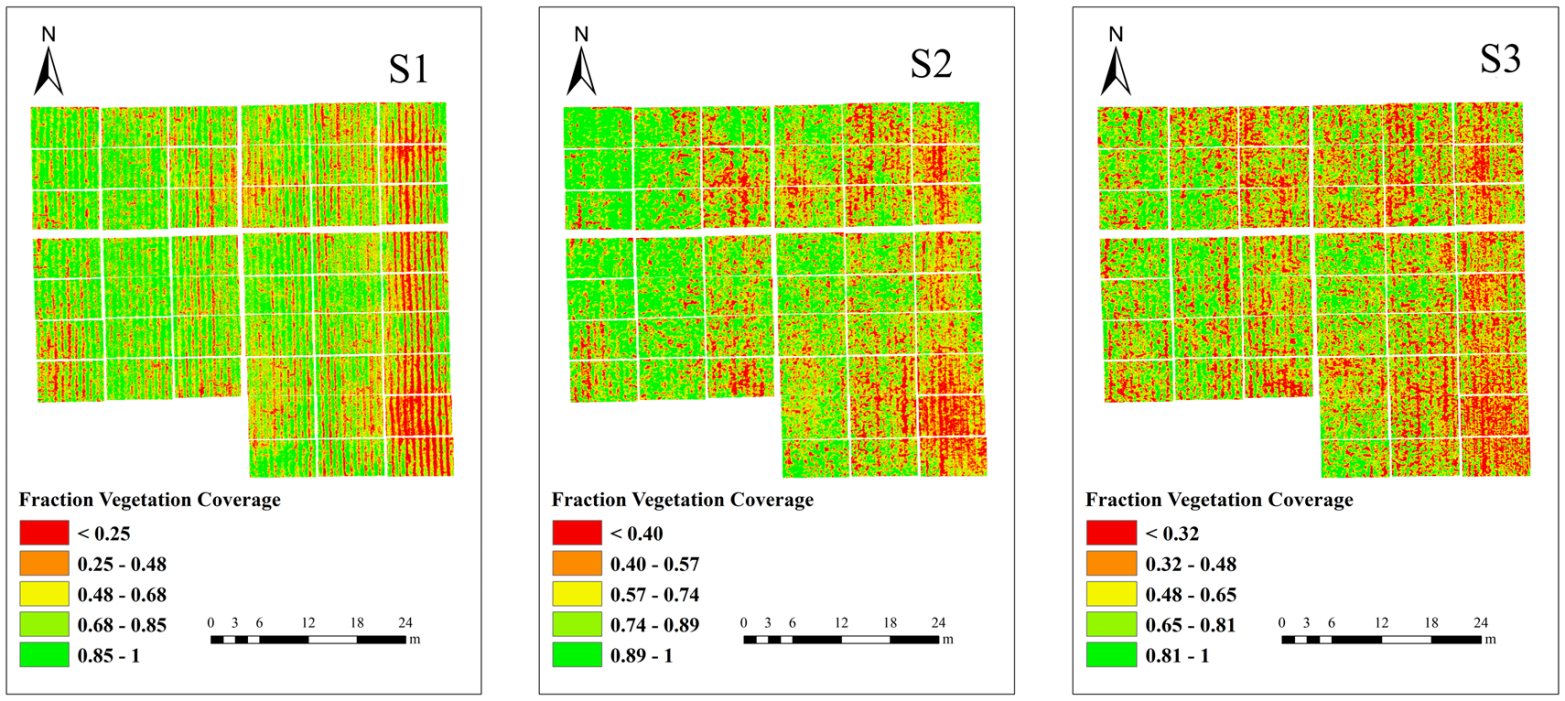
FVC extraction results.

**Figure 6:**
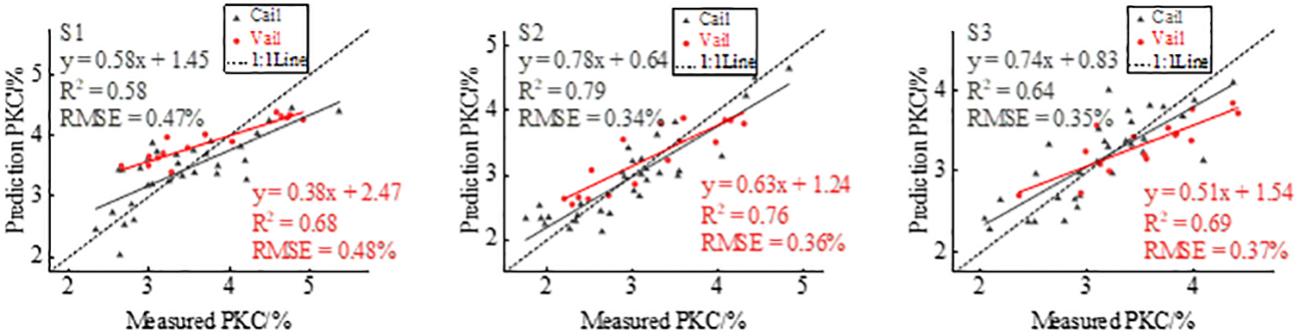
GNDVI combined with FVC to estimate potato PKC.

**Figure 5** illustrates a pattern in potato FVC, where it initially increases and then decreases as the growth stages progresses. Based on the multispectral VIs used in this study, the optimal VIs were selected and combined with FVC to estimate potato PKC using SMLR modeling, and the results are shown in **Figure 6**. The modeling *R* ^2^ for the three fertility periods is 0.58, 0.79, and 0.64, and the RMSE is 0.47%, 0.34%, and 0.35%, respectively. The *R* ^2^ of the validation set was 0.68, 0.76, and 0.69, and the RMSEs were 0.48%, 0.36%, and 0.37%, respectively. The modeled and validated *R* ^2^ of potato PKC increased in all three growth stages compared with that of GNDVI only, with the most significant increase in the starch accumulation stage and the decrease in the RMSEs of tuber growth and starch accumulation stages. The SMLR model performs best at the tuber growth stage (**Table 5**).

**Table 5:** Estimation of potato PKC modeling and verification analysis with different model parameters in three growth periods.

		S1		S2		S3	
		*R* ^2^	RMSE (%)	*R* ^2^	RMSE (%)	*R* ^2^	RMSE(%)
VIs	Calibration	0.56	0.47	0.68	0.43	0.51	0.40
	Validation	0.53	0.58	0.74	0.39	0.58	0.42
VIs+FVC	Calibration	0.58	0.47	0.79	0.34	0.64	0.35
	Validation	0.68	0.48	0.76	0.36	0.69	0.37
VIs+RDTI	Calibration	0.62	0.44	0.75	0.37	0.55	0.39
	Validation	0.77	0.54	0.81	0.37	0.71	0.24
VIs+FVC+RDTI	Calibration	0.63	0.44	0.84	0.29	0.80	0.25
	Validation	0.78	0.36	0.86	0.30	0.80	0.35

#### 3.3.2 Multispectral VIs combined with texture features for estimating potato PKC

**Figure 7:**
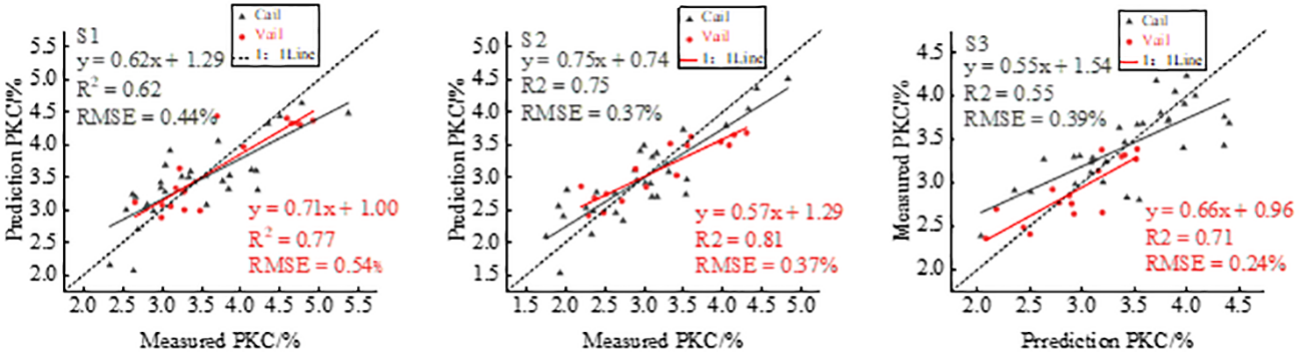
GNDVI combined with RDTI estimation of potato PKC.

Since the correlation between RDTI and potato PKC based on the texture feature combinations extracted from GLCM with different orientations was not significant in relation to the fertility stage and was comparable to the correlation with multispectral VIS, in this study, we chose the texture feature combinations in the 45° orientation to screen out the RDTI with the highest absolute value of correlation coefficient with potato PKC and GNDVI to estimate potato PKC, and each fertility stage was used to the single texture features used to construct the optimal RDTI were different for each fertility stage, including Green-Mean and Blue-Mean for S1 stage, Red_Con and Reg_Var for S2 stage, and Red-Mean and Green-Dis for S3 stage, and the results of the estimation are shown in **Figure 7**. The modeled *R* ^2^ for the three fertility periods is 0.62, 0.75, and 0.55, and the RMSE is 0.44%, 0.37%, and 0.39%, respectively. The SMLR model performed best in the tuber growth stage (**Table 5**).

#### 3.3.3 Multispectral VIs combined with FVC and texture features for estimating potato PKC

**Figure 8:**
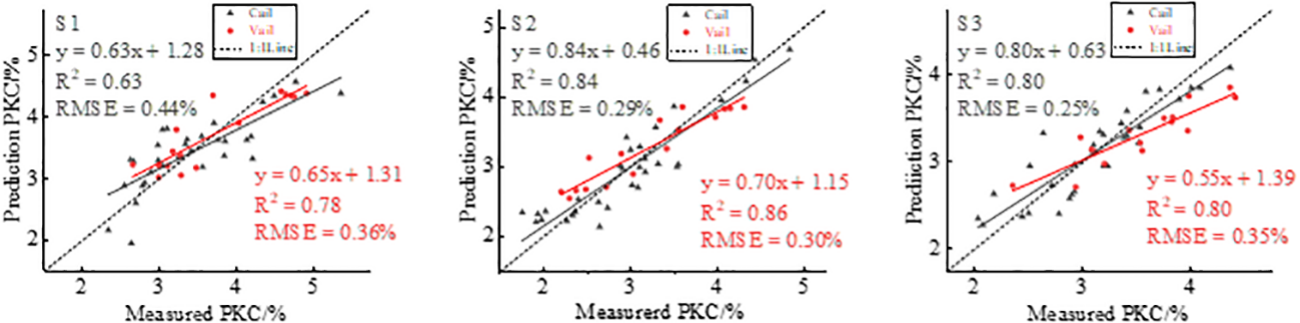
GNDVI combined with FVC and texture features for estimating potato PKC.

Based on the optimal VIs selected in this study, the extracted FVC and RDTI were used to estimate potato PKC using SMLR modeling. **Figure 8** shows the results of the fusion modeling of GNDVI, FVC, and RDTI to estimate potato PKC. The modeling *R* ^2^ is 0.63, 0.84, and 0.80, respectively, and the RMSE is 0.44%, 0.29%, and 0.25%, respectively. The validation *R* ^2^ is 0.78, 0.86, and 0.80, and the RMSE is 0.36%, 0.30%, and 0.35%, respectively. The modeling and validation *R* ^2^ values are greater than those estimated from GNDVI, GNDVI combined with FVC, and GNDVI combined with RDTI for potato PKC over the three growth stages, whereas the RMSE was lower than those estimated using GNDVI, GNDVI combined with FVC, and GNDVI combined with RDTI. GNDVI was combined with RDTI to estimate the RMSE of potato PKC, and the SMLR model has the highest accuracy in the tuber growth phase (**Table 5**).

## 4 Discussion

### 4.1 Effect of different experimental treatments on PKC and canopy structure of potato

The experimental design used in this study encompasses various gradients of potassium fertilizer dosage, plant density, and nitrogen fertilizer dosage, enabling an investigation of their effects on the growth and development of potato plants. The findings reveal distinct patterns in potato PKC across the growth stages advance, consistent with the research conducted by Liu Keli et al. (Liu et al., 2003). During the tuber formation stage, potato plants are in an early developmental phase, and applying fertilizer contributes to a higher PKC. This is attributed to the fact that the plants were not yet fully developed, so the nutrients supplied through fertilization had a pronounced impact on PKC during this stage. As the potato plants progress to the late tuber formation and early tuber growth stages, rapid growth occurs primarily through stem and leaf expansion, whose dilution affects the PKC. Consequently, the PKC gradually decreases during this phase. However, during the late tuber growth period, the aboveground growth of potato plants, including stems and leaves, reaches its peak, and the rate of growth begins to slow. At this stage, the potato plants transition from pure nutrient-driven growth to a combination of nutrient assimilation, reproductive growth, and material accumulation. This phase represents the peak of potato growth and development. Importantly, nutrients relocate from aboveground parts to the underground tubers, resulting in the lowest PKC levels during the tuber growth period (Liu et al., 2022a).

This study used a selection of nine multispectral VIs relevant to potato PKC and analyzed their correlation with PKC at three crucial growth stages. As depicted in **Figure 3**, all VIs correlate significantly with PKC at a significance level of 0.01, which reflects the suitability of the selected multispectral VIs for estimating PKC in potatoes. The correlation between multispectral VIs and PKC initially increases, followed by a decrease from the tuber formation stage to the starch accumulation stage. The results of potato PKC estimation using multispectral VI modeling (**Table 4**) reveal that the most accurate PKC estimation occurs during the tuber growth stage. This result is attributed to the inherent mechanisms governing potato growth, development, and fertilization. During the tuber formation and tuber growth stages, potato plants undergo vigorous growth, and the extracted canopy spectra are less influenced by soil interference, allowing the VIs to more accurately reflect the PKC. However, in the late growth period, as potato plants start to senesce and yellow, the extracted canopy spectra become more susceptible to soil background effects, leading to a less accurate representation of PKC. Additionally, spectral saturation during this stage reduces the VI sensitivity to PKC, lowering the estimation accuracy. Orientation does not affect the correlation between the MEAN and RDTI constructed based on GLCM texture and potato PKC under a 3×3 window. Generally, the MEAN texture feature correlates better with potato PKC, and the constructed RDTI correlates more strongly compared with individual texture features. This result is explained by the smallest window size being the best to capture canopy texture variations at different potato growth stages. Furthermore, the smaller window size avoids exaggerating differences within the window or excessively smoothing texture variations (Zheng et al., 2019). The MEAN texture feature, which represents the average of target and background moving windows, contributes to image smoothing and reduces interference from the soil background (Zheng et al., 2019). By combining the advantages of normalized VIs and difference VIs, RDTI effectively mitigates the effects of soil background, solar altitude angle, and azimuth while enhancing the correlation with potato PKC by smoothing the canopy structure (Haboudane et al., 2004; Eckert, 2012).

### 4.2 VIs combined with spatial structural features to estimate potato PKC

The transition in canopy structure from simple to complex during the potato tuber formation and starch accumulation stages corresponds to changes in the FVC with fertility. Morphological parameters have been widely used for monitoring the physicochemical parameters of crops (Bendig et al., 2015; Stevens et al., 2020). However, the relationship between morphological parameters and potato PKC at different growth stages remains unclear. From tuber formation to the initial stage of tuber growth, potato growth is primarily characterized by stem and leaf development, which gradually reaches completion. Consequently, the FVC gradually increases, reaching a saturation point after which it no longer changes significantly (Wan et al., 2020; Qiao et al., 2022). During the late tuber growth and starch accumulation stages, the FVC decreases due to the gradual transfer of nutrients from aboveground to below-ground parts of the plant. Additionally, aboveground stems and leaves begin to wither and yellow. These observations suggest that the FVC may be associated with potato PKC. To investigate whether the FVC can enhance the accuracy of estimating potato PKC, we extracted the FVC at three different potato growth stages using the image dichotomy method. The best-performing VIs were then fused to model potato PKC. The highest model accuracy was observed during the tuber growth stage. This result can be attributed to the close correspondence between potato growth and nutritional status during this stage, leading to more accurate extracted FVC values. Conversely, the starch accumulation stage is characterized by reproductive growth, with potassium continuously being transferred to the tuber. As a result, the accuracy of the extracted FVC values diminishes during this stage.

The changes in potato canopy structure, which are influenced by fertility variations, also result in variations in the extracted GLCM textures. Specifically, Green_Con, Red_Con, Reg_Con, and Nir_Con correlate to various extents with potato PKC and fertility, suggesting their association with canopy structure. Previous studies on wheat and maize have shown a monotonic increase in texture characteristics throughout the reproductive period (Yue et al., 2018; Zhu et al., 2021). However, in the case of potato canopy texture, an increasing trend followed by a decreasing trend occurs (Liu et al., 2022b), which implies that potato canopy texture characteristics correlate with potato PKC. Comparing **Figures 4**, **7** shows that integrating VIs with RDTI in the modeling and validation stages increases *R* ^2^ and decreases RMSE across all three fertility periods. The highest model accuracy occurs during the tuber growth period. Furthermore, comparing **Figures 6**, **7** reveals that, during the tuber formation stage, the accuracy of VIs combined with RDTI is superior to that of VIs combined with the FVC. Conversely, the accuracy of VIs combined with FVC is greater than that of VIs combined with RDTI during the tuber growth stage. This discrepancy is attributed to the influence of soil background on the accuracy of FVC extraction during the tuber formation stage. However, RDTI effectively mitigates the interference caused by the soil background. The tuber growth period represents the peak of potato growth and development, where the canopy structure is less affected by the soil background. Consequently, the extracted FVC more accurately reflects the potassium nutrient status of the potato.

The statistical analysis conducted in this study demonstrates that integrating multispectral VIs with FVC or texture features enhances the accuracy of potato PKC estimates compared with using multispectral VIs alone. This finding underscores the significance of incorporating FVC and texture features for accurate potato PKC estimation. The results presented in **Figure 8** indicate that the combination of multispectral VIs with FVC and texture features further improves the accuracy of potato PKC estimation. Multispectral VIs contribute valuable spectral information about the potato canopy, while FVC provides essential structural information. Additionally, texture features offer high-frequency information pertaining to the canopy. Integrating these diverse sets of information provides a more comprehensive understanding of potato PKC variation. This fusion approach facilitates the capture of complementary details regarding potato PKC and enables a more comprehensive assessment of its variations.

### 4.3 Strengths and weaknesses of this study

This study shows that the fusion of multispectral VIs with morphological parameters and texture features enhances the accuracy of potato PKC estimation. This finding is consistent with previous research, which found that the fusion of spectral VIs with morphological parameters and texture features improves the accuracy of estimating biomass (Yue et al., 2018; Zheng et al., 2019), the leaf area index (Li et al., 2019), and nitrogen (Zheng et al., 2020; Fan et al., 2022). The SMLR model, which combines the strengths of spectral features, morphological parameters, and texture features, proves advantageous in improving potato PKC estimation accuracy. Moreover, this model is particularly suitable for agricultural managers with limited budgets who seek to utilize multispectral sensors. Note that multispectral sensors may not be ideal for extracting morphological parameters and texture features due to their lower spatial resolution. In this study, only one crop and one potato location were examined over a single year. Therefore, validating the model across different locations, crops, and years is essential. Although the UAV platform used in this study is suitable for small-scale operations, it is susceptible to flight instability caused by wind and flight velocity. Additionally, its limited endurance creates difficulties for large-scale monitoring of crop physicochemical parameters. However, with the rapid advancement of UAV technology, commercial fixed-wing UAVs now offer longer flight durations (e.g., X-1 Chimera, 4). As a result, the method proposed herein for monitoring the potassium nutrient status in crops provides valuable insights and reference value for future applications.

## 5 Conclusion

This study proposes a method for monitoring potato PKC at critical growth stages using UAV-based multispectral sensors. The study focuses on extracting canopy spectral features, FVC, and GLCM texture features from multispectral images of potatoes during critical fertility periods. The study aimed to explore how combining VIs with FVC, VIs with GLCM texture features, and the fusion of VIs, FVC, and GLCM texture features affect the accuracy of potato PKC estimation when using the SMLR model. The results lead to the following conclusions: (1) The accuracy of estimating potato PKC using VIs extracted from multispectral images alone was moderate. Among the VIs tested, the GNDVI performed the best, with modeling *R* ^2 = ^0.56, 0.68, and 0.51 and RMSE = 0.43%, 0.43%, and 0.40% for the three fertility periods of potato tuber formation, tuber growth, and starch accumulation, respectively. (2) Combining GNDVI with FVC during the three fertility periods improved potato PKC estimation accuracy. The modeling *R* ^2^ values are 0.58, 0.79, and 0.64, and the corresponding RMSE values are 0.47%, 0.34%, and 0.35% for the three fertility periods of potato tuber formation, tuber growth, and starch accumulation, respectively. (3) The RDTI, composed of two random textures, correlates with potato PKC more than with single texture features. Combining GNDVI with RDTI also enhanced the accuracy of estimating potato PKC. The modeling R^2^ values for the three fertility periods are 0.62, 0.75, and 0.55 and the corresponding RMSE values are 0.44%, 0.37%, and 0.39%, respectively. (4) The fusion of GNDVI, FVC, and RDTI further improves the accuracy of the SMLR model for estimating potato PKC. The resulting *R* ^2^ values for the three fertility periods are 0.63, 0.84, and 0.80 and the corresponding RMSE values are 0.44%, 0.29%, and 0.25%, respectively. Overall, this study provides valuable insights into using UAV-based multispectral sensors for monitoring the potassium nutrient status of crops. These findings contribute to reducing agricultural production costs and enhancing precision agricultural management practices. Further research is needed to validate the proposed method for different locations, crops, and years to ensure its applicability in diverse agricultural settings.

## Data availability statement

The original contributions presented in the study are included in the article/supplementary material. Further inquiries can be directed to the corresponding authors.

## Author contributions

YM: Writing – original draft, Writing – review & editing. ZC: Writing – review & editing. YF: Writing – review & editing, Investigation, Methodology, Project administration. MB: Writing – original draft, Writing – review & editing, Conceptualization, Data curation, Formal Analysis. RC: Writing – original draft, Writing – review & editing, Investigation, Methodology, Software, Validation. GY: Writing – original draft, Writing – review & editing, Methodology, Software, Supervision, Visualization. HF: Writing – original draft, Writing – review & editing.
